# Individualized prophylaxis for optimizing hemophilia care: can we apply this to both developed and developing nations?

**DOI:** 10.1186/s12959-016-0096-y

**Published:** 2016-10-04

**Authors:** Man-Chiu Poon, Adrienne Lee

**Affiliations:** 1 Department of Medicine, Cumming School of Medicine, University of Calgary, Calgary, Alberta Canada; 2 Department of Pediatrics, Cumming School of Medicine, University of Calgary, Calgary, Alberta Canada; 3 Department of Oncology, Cumming School of Medicine, University of Calgary, Calgary, Alberta Canada; 4 Southern Alberta Rare Blood and Bleeding Disorders Comprehensive Care Program, Foothills Hospital, Alberta Health Services, Calgary, Alberta Canada

**Keywords:** Hemophilia, Individualized prophylaxis, Personalized prophylaxis, Pharmacokinetics, Population pharmacokinetics, Low-dose prophylaxis, Terminal half-life

## Abstract

Prophylaxis is considered optimal care for hemophilia patients to prevent bleeding and to preserve joint function thereby improving quality of life (QoL). The evidence for prophylaxis is irrefutable and is the standard of care in developed nations. Prophylaxis can be further individualized to improve outcomes and cost effectiveness. Individualization is best accomplished taking into account the bleeding phenotype, physical activity/lifestyle, joint status, and pharmacokinetic handling of specific clotting factor concentrates, all of which vary among individuals. Patient acceptance should also be considered. Assessment tools (e.g. joint status imaging and function studies/scores, QoL) for determining and monitoring risk factors and outcome, as well as population PK profiling have been developed to assist the individualization process. The determinants of optimal prophylaxis include (1) factor dose/dosing frequency, hence, cost/affordability (2) bleeding triggers (physical activity/lifestyle, chronic arthropathy and synovitis) and (3) bleeding rates. Altering one determinant results in adjustment of the other two. Thus, the trough level to protect from spontaneous bleeding can be increased in patients who have greater bleeding risks; and prophylaxis to achieve zero joint bleeds is achievable through optimal individualization. Prophylaxis in economically constrained nations is limited by the ill-affordability of clotting factor concentrates. However, at least 5 studies on children and adults from Thailand, China and India have shown superiority of low dose (~5–10 IU kg^−1^ 2-3× per week) prophylaxis over episodic treatment in terms of bleed reduction, and quality of life, with improved physical activity, independent functioning, school attendance and community participation. In these nations, the prophylaxis goals should be for improved QoL rather than “zero bleeds” and perfect joints. Prophylaxis can still be individualized to affordability. Higher protective trough level can be achieved by using smaller doses given more frequently without an increase in consumption/cost. The bleeding trigger can also be down-regulated by avoiding unnecessary injury, and by engaging in judicious strengthening exercises appropriate to the joint status to improve balance and joint stabilization. Central to the success of prophylaxis are clinics with comprehensive care that provide the necessary professional expertise, support, and counseling, to educate patients, families, and other healthcare professionals, and to support research for improved hemophilia care.

## Background

Individualized prophylaxis has become an important topic in how prophylaxis can be optimized based on various patient factors such as bleeding risk, pharmacokinetic (PK) profile, joint status and physical activity/lifestyle. With the understanding of interpatient heterogeneity influencing these factors, individualized prophylaxis should be the ideal strategy to optimize factor utilization while improving patient quality of life (QoL) and joint health. In developed nations where there are fewer constraints in terms of factor affordability, the ultimate goal of “zero bleeds” and possibly perfect joints is achievable with the right peak and trough levels adjusted for individual risk factors and PK handling. Assessment tools (e.g. MRI/ultrasound imaging for joint status, QoL tools, joint scores) for determining and monitoring risk factors and outcome, as well as population PK profiling have been developed to assist us in this individualization process. Although prophylaxis in general is expensive, and the proposed tools to assist with individualization are time consuming and resource intensive, the overall economic savings from improved QoL with almost no bleeding and bleed-related complications, should be more than enough to offset the costs of its implementation in resource adequate nations. The question is whether it is possible for the developing world to practice individualized prophylaxis when there are barely enough resources and affordable factor concentrate to ensure all bleeds are treated adequately or even treated at all. Approximately 75–80 % of the world’s hemophilia population lives in developing nations and we are at risk of leaving this large proportion of patients behind and undertreated if we assume the goal for prophylaxis should always be “zero bleeds”. Prophylaxis regimens that focus on using resource intensive measurement tools, and PK profiling models may not be attainable to all. However, this does not mean developing nations should not strive to achieve some form of prophylaxis in the interim. Simply the goals of prophylaxis need to be adjusted such that it is individualized for QoL rather than “zero bleeds”, for independent function and gainful employment rather than pristine joints, and for trough levels that minimize bleed events rather than PK profiling to ensure a certain pre-defined trough level. For resource-limited countries, prophylaxis must be individualized to include affordability as a major determinant in order to optimize low-dose regimens that have demonstrated superiority compared to episodic treatment. This review aims to communicate the current concepts of individualized prophylaxis and how this might be adapted and applied in both developed and developing nations.

## Review

### The evolution of prophylaxis

Prophylaxis is considered optimal care for hemophilia patients to prevent bleeding and to preserve joint function [1]. The original idea being that keeping factor levels above 1 % converts severe hemophilia to a moderate severity phenotype where spontaneous joint bleeds and chronic arthropathy is less frequently observed. The Swedish have been practicing prophylaxis in young boys since the 1960’s and demonstrated continuous prophylaxis started at an early age to prevent factor levels from falling below 1 %, preserved joint function, and allowed patients to lead normal lives [2]. The superiority of primary prophylaxis (for definition, see Table 1) compared to episodic treatment is no longer questioned based on pivotal randomized controlled studies [3, 4]. Studies of secondary and tertiary prophylaxis in adolescents and adults have also shown benefit in reducing annual bleeding rate (ABR), rate of joint deterioration, and number of days lost from school or work compared to episodic treatment [5–7] With this irrefutable evidence, prophylaxis (over episodic treatment) should be the standard of care to all severe hemophilia individuals.

**Table 1:** Definitions of continuous prophylaxis (see reference [1])

	No. large joint bleeds*	Age to start (year)	Clinical arthropathy, osteochondral disease
Primary	≤1	≤3	absent
Secondary	≥2	any	absent
Tertiary	≥2	any	present

All refer to continuous prophylaxis intended for 52 weeks per year, and taken for at least 45 weeks of the year under consideration

*large joints =  knees, ankles, elbows, hips, shouders

However, practical issues related to the cost and burden of prophylaxis, together with the recognized heterogeneity in bleeding risk and PK handling of factor concentrates, have lead us to question what the ideal prophylaxis regimen for severe hemophilia should be. High-dose (Malmö) prophylaxis regimen is considered “gold standard” [2]. Unfortunately, the costs associated with such a regimen and patient acceptance of frequent infusions starting at a very young age has been prohibitive for its widespread implementation. Alternatively, intermediate-dose prophylaxis has been used by the Dutch since 1968 (Table 2) [8]. A long-term outcome study that compared the high-dose Malmö to the intermediate-dose Dutch prophylaxis showed comparable QoL (EQ-5D 0.84 vs 1.0), despite a small but significant reduction in median annual joint bleeding rate (AJBR, 1.3 vs. 0) and hemophilia joint health score (HJHS, 9 vs 4) [8]. However, annual factor consumption was 2150 IU kg^−1^ per year lower for the Dutch regimen resulting in a sizeable cost difference (US$159,000 based on US$1.10 per unit [8]) that argued against cost-effectiveness for quality-adjusted life years (QALY) for high-dose prophylaxis.

**Table 2:** Primary prophylaxis regimens

	Dosing
High/full-dose (Malmö/Swedish) [2]	25–40 IU kg^−1^ 3 times a week or every other days starting at age 1–2 years, irrespective of bleeding history
Intermediate-dose (Dutch) [8]	15–25 IU kg^−1^ 2–3 times per week, usually started after ≥1 hemarthrosis
Escalating-dose (Canadian) [9]	50 IU kg^−1^ once a week, with dose increased to 30 IU kg^−1^ twice a week, then 25 IU kg^−1^ every other day, in response to bleeding frequency

The Canadian Hemophilia Primary Prophylaxis Study (CHPS) looked at reduced-intensity escalating dose prophylaxis (Table 2) [9]. The impetus for this regimen followed observations that about 10–15 % of severe hemophilia patients have infrequent bleeding and hemarthroses, and little or no joint damage [10]. The CHPS study aimed to tailor the prophylaxis regimen to balance the burden of IV injections, need for central venous catheter (CVC) insertions, and the costs of factor concentrate, against the level of arthropathy and patient’s QoL. In this study, patients were able to preserve reasonable joint function with a mean AJBR of 0.78. At 15 years follow up 9 % of subjects remained on step one (50 IU/ kg^−1^ once weekly prophylaxis) and 32 % on step two (30 IU kg^−1^ twice weekly) [9]). The mean annual factor consumption was 3228 IU kg^−1^ per year, less than the ~ 6000 IU kg^−1^ per year for full-dose alternate day regimen [11]. In addition only 30 % of subjects in CHPS required CVCs compared to 82 % from a survey of North American centres of children on full-dose prophylaxis [12]. A cost-utility analysis using Markov modelling taking into account costs of factor, medical resources, effectiveness and health-related QoL (HR-QoL) showed the QALY for full-dose and escalating dose (CHPS) prophylaxis were the same due to the trade-off in greater number of CVCs needed for full-dose and the higher number of bleeds with escalating dose prophylaxis. However, the incremental cost per QALY gained with full-dose was > CAD$1,000,000, suggesting escalating dose prophylaxis is the greater cost-saving strategy of the two regimens [13].

### The shift in paradigm: individualized prophylaxis

CHPS was a pioneer in tailoring prophylaxis and was able to produce adequate preservation of joint status using less factor with some cost saving. However, CHPS takes into account only one aspect of bleeding risk, bleed frequency, and its applicability is limited to pediatric patients initiating primary prophylaxis. Individualizing prophylaxis that is applicable to all age groups should take into consideration additional factors that contribute to bleeding risk such as level of physical activity/lifestyle, existing joint arthropathy, chronic synovitis, and time spent below an acceptable trough level, as well as patient’s acceptability, burden of frequent venipunctures and venous access issues especially in children, and ability to self-guided care. The intrinsic half-life of a factor concentrate and the variation in patient PK handling influences the strategies for individualizing prophylaxis. This has generated intense interest in measuring an individual’s PK profile, particularly elimination half-life of factor concentrates for an individual patient. Full PK profiling requires measuring multiple time points following factor infusion to generate an accurate estimate of elimination half-life. To circumvent the inconvenience of multiple venipunctures, population PK models have been developed for various factor concentrates to predict individual PK parameters with only 2 or 3 time points. Understanding a patient’s PK profile allows tailoring a prophylaxis regimen to a dose and interval that achieves a predetermined optimal trough level that will minimize bleeding and maximize cost effectiveness for that particular patient.

What target trough level should be used has been an issue of debate. It is widely accepted that keeping trough levels above 1 % should be a minimum as few joint bleeds occur above this level. However, there is emerging evidence to suggest that maybe 1 % is not sufficient and that targeting higher trough levels should be pursued to maintain the healthiest joints possible. Epidemiologic evaluation on a Dutch cohort at diagnosis demonstrates that a FVIII level at 1 % may still have upwards of 5 joint bleeds per year, while levels >10–12 % have essentially zero joint bleeds [14]. It has been argued that even one bleed into the healthy joint of a growing child is one too many [15]. Canine studies demonstrate that a single joint bleed produces enough inflammation to cause permanent damage to developing cartilage. It is possible that the same in a maturing human joint may have deleterious effects as the individual ages [16]. We currently have limited long term data on primary prophylaxis regimens with the most mature data coming from the Swedish versus Dutch prophylaxis study that supports the idea that higher troughs achieve the healthiest joints due to near zero AJBR [8]. Additionally the US joint outcome study showed MRI-detectable joint damage still occurred in individuals who had no clinically evident joint bleeding [4]. Presumably, this resulted from subclinical joint bleeds which theoretically may be averted by a prophylaxis regimen that maintained higher trough levels.

### Determinants of individualized prophylaxis

**Fig. 1:**
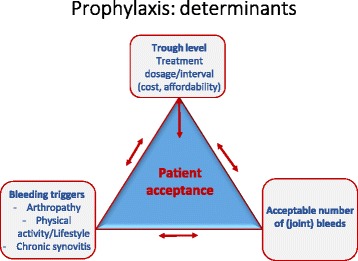
Determinants of prophylaxis. Prophylaxis treatment regimen has 3 main determinants: (1) the given resources/concentrate availability/afffordability to target a specific trough level and/or dosage/intervals of infusions, all of which reflect the consumptions/costs; (2) the bleeding trigger, which comprises physical activity/lifestyle, presence and degree of arthropathy, and presence of chronic synovitis; and (3) the number of bleeds, especially joint bleeds, that are regarded as acceptable. These 3 determinants form a triangle. If 1 determinant is changed, the other 2 will adjust. Central to these, patient acceptability and ability to self-guided care must also be considered. With unlimited resources, “zero bleeds” and normal physical activity may be targeted. With few resources, only low-dose substitutions may be given, thus accepting a certain number of bleeds and limited physical activity. None-the-less where acceptable to the patient with adequate venous access, increasing frequency of infusion will attain a higher trough level with a lower dose (and hence consumptions/costs, see Fig. 2). Determinant 2 can also be improved by avoiding injury and by improving muscle strength and balance with exercise appropriate to the patients’ joint status. (Adapted from Oldenburg [17], with permission)

In a recent review on optimizing prophylaxis [17], Oldenburg proposed 3 main determinants for prophylaxis (Fig. 1): (1) a target trough level dictated by dose and interval of prophylaxis based on patient PK parameters and the availability and cost of factor concentrate, (2) the bleeding triggers which includes degree of physical activity/lifestyle, presence and severity of joint arthropathy and chronic synovitis, and (3) the number of bleeds, specifically joint bleeds, that are deemed acceptable (Fig. 1). These 3 factors form a dynamic triangle for optimizing prophylaxis. When 1 determinant is changed, the other 2 will adjust. For example, in the setting of unlimited resources “zero bleeds” and normal or even intense physical activity is achievable by using high dose, frequent injections to target higher troughs (5–10 % or more). At the centre, patient acceptance and ability to self-guided care will play a role in how much weight can be placed on each corner of this triangle.

In the developed world where affordability of factor is not a major constraint, determinant (1), has a proportionally greater influence on the triangle since the trough level is the determinant we have the greatest control over. For determinant (2), bleeding triggers such as degree of arthropathy, presence of chronic synovitis, are inherent to the individual and cannot be easily changed. However, physical activity/lifestyle can be adapted to the degree of arthropathy, and the leeway to intensify physical activity is driven by trough level and number/intensity of peaks that determinant (1) can afford. Determinant (3), number of bleeds, is then a direct consequence of mostly how much determinant (1) can be changed. Therefore, in the developed world prophylaxis regimens should be adjusted to achieve the lowest number of bleeds possible. Given that the terminal half-life has the greatest impact on the trough, simply shortening the interval between infusions, even by 24 h, will cumulatively increase the trough level. Recent introduction of extended half-life products will provide patients with more options on frequency of infusion for a desired trough level.

### Prophylaxis in resource limited nations: using low-dose and how it can still be individualized

The determinants of prophylaxis remain the same for developing nations, however; the freedom to adjust the determinants is impaired by lack of resources and affordable product. Thus, the majority of patients in developing countries continue to receive episodic treatment rather than prophylaxis. Full dose, and even intermediate dose prophylaxis is clearly not affordable/possible. Thus, adjusting trough levels to achieve a predefined target may become a moot point in the prophylaxis triangle. Without prophylaxis, these patients suffer tremendous disability, leaving them with crippling arthropathy, and unable to integrate fully to society. Many become wheelchair bound, unable to attend school or secure gainful employment.

It seems unacceptable to deny 75–80 % of the world’s hemophilia population of prophylaxis when the benefits of this treatment are so clear. However, studies from Thailand, China and India are providing evidence that even low-dose prophylaxis can have major impact on number of bleeds, QoL, and functional participation in society. Four pediatric pilot studies, 3 from Thailand and China, and a small randomized study from India, used low-dose prophylaxis 8–10 IU kg^−1^ twice a week. Even though the numbers were small and the follow up ≤1 year, these studies demonstrate significant reductions in ABRs, AJBRs, fewer days absenteeism from school, and improved QoL despite only 37 % of children in the Indian study had measured trough levels ≥1 % [18–21]. Factor consumption with prophylaxis was higher compared to on demand (1050 vs 675 IU kg^−1^ per year) in the Indian study [21] but is significantly lower when compared to full dose (~6000 IU kg^−1^ per year) [11] and escalating dose (~3228 IU kg^−1^ per year) [9] prophylaxis.

Only one study has looked at low-dose tertiary prophylaxis in adult patients who at baseline already have established severe arthropathy. This Chinese study used 5–10 IU kg^−1^ 2–3 times per week depending on what the patient can afford, and demonstrated a 77 % reduction in ABR and significant improvement in functional independence scores (FISH) [22]. Although there was no measurable structural improvement (unchanged radiologic joint scores), some of the wheelchair-bound patients were able to walk on their own again, highlighting the significant impact low-dose prophylaxis made. Although solid evidence from large clinical trials on the benefits of low-dose prophylaxis is still lacking, it appears to be a feasible way to deliver prophylaxis treatment in resource-limited nations; at least until higher dose prophylaxis becomes economically possible.

**Fig. 2:**
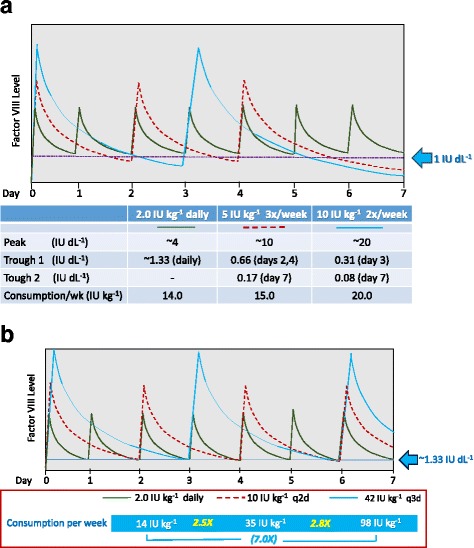
Low dose prophylaxis in economically constrained environment: Influence of FVIII infusion frequency on trough level and factor consumption. (Modeled based on an average FVIII recovery of 2 IU dL^−1^ per IU kg^−1^ infused and a T_1/2_ of 12 h). **a** A dosage of 10 IU kg^−1^ two-times a week as well as 5 IU kg^−1^ three-times a week (weekly consumption 20 and 15 IU kg^−1^ per week respectively) each results in a trough level <1 IU dL^−1^ in 3–4 of the 7 days in the week (but with trough levels always higher with three-times a week than with two-times a week prophylaxis even at lower dose with lower consumption), whereas as little as 2 IU kg^−1^ daily (qd, weekly consumption 14 IU kg^−1^ per week) produces daily trough of ~1.33 IU dL^−1^. [For three-times weekly prophylaxis, doubling the infusion dose from 5 to 10 IU kg^−1^ will double the day 2, 4 and 6 trough levels to ~1.33 IU dL^−1^, but still leave trough level on day 7 at 0.66 IU dL^−1^ (i.e. <1 IU dL^−1^, figure not shown)]. Prophylaxis at 10 IU kg^−1^ every-two-day (q2d) is shown in Fig. 2b. **b** In order for an every-three-day (q3d) regimen to produce a trough level similar to that obtained by every-two-day (q2d) infusion (e.g.1.33 IU/dL), the dosage per infusion has to be increased, whereas daily infusion requires a lower per infusion dosage. Compared to the q2d regimen, factor consumption is 2.8× more for the q3d regimen but 2.5× less for the qd regimen. These relative consumption multiples are the same for other target trough levels and other PK handling of clotting factors for a particular individual. (Figures not drawn to scale. Peak and trough levels will be different for different patients depending on their individual pharmacokinetic handling of the particular clotting factor, but the principles remain the same. Peak and trough levels tend to increase slightly with infusions but remain more or less constant after the first few infusions and steady state is achieved. Values for peak levels represent value range during steady state with each regimen)

As use of low-dose prophylaxis begins to gain support and is implemented in other resource-limited countries; we believe that the principles of individualization can still be used to optimize prophylaxis for any given patient within their fiscal limitations to maximize benefits. By determining how much factor a patient can afford over a given period of time, much more effective prophylaxis with higher trough levels can be achieved for that individual by giving smaller doses more frequently (Fig. 2). Figure 2a shows how increasing dose frequency can improve troughs without increasing consumption or costs. Assuming an average FVIII recovery of 2 IU dL^−1^ per IU kg^−1^ infused and a T_1/2_ of 12 h, a dosage of 10 IU kg^−1^ two-times a week or 5 IU kg^−1^ three-times a week (weekly consumption 20 and 15 IU kg^−1^ per week respectively) each results in a trough level <1 IU dL^−1^ in 3–4 out of the 7 days per week, whereas as little as 2 IU kg^−1^ daily (qd, weekly consumption 14 IU kg^−1^ per week) produces daily troughs of ~1.33 IU dL^−1^. This is an example of adjusting determinant (1) to individualize prophylaxis based on affordability in environments of resource constraint. This is particularly important for older children and adults with higher weight (hence, total dose) but have good veins for more frequent infusion. Conversely, to maintain any given trough level (e.g. 1.33 IU dL^−1^), clotting factor consumption per week is decreased by ~2.5× when given daily, and increased by ~2.8× when given every 3^rd^ day in comparison to “standard” every-other-day prophylaxis (Fig. 2b).

In order for prophylaxis to work in developing nations, there must be a clinic where by injections are given or patients are taught to self-inject (home care), and education for self-care and prophylaxis acceptance/adherence can be provided. Clinic support is also needed to educate patients on appropriate physical activity and life style adjustment, bleeding triggers that can be controlled. Hemophilia patients in developing nations often shy away from exercising because of the fear of bleeding. However, proper education on preventing avoidable injuries (including use of protective gear) and exercises that are appropriate to their joint status and degree of arthropathy requires professional help from trained-physiotherapists and physicians. Exercises to improve muscle strength and balance help stabilize joints and can avert joint bleeds in patients who are less protected on low dose prophylaxis. A dedicated hemophilia clinic with comprehensive care remains fundamental to prophylaxis irrespective of economic capacity, and should also be the priority in all developing nations.

## Conclusion

Prophylaxis has significantly changed the lives of many hemophilia patients. Those living in developed nations have already benefited from prophylaxis, but room remains for improvement. Individualized prophylaxis by understanding bleeding triggers and PK profiles would allow us to target appropriate trough levels that can achieve “zero bleeds”, provide the greatest chance of preserving joint health, and ultimately meet the specific goals for each patient. Although prophylaxis is expensive, this is not a reason for developing nations to remain complacent with inferior episodic treatment. Low and very-low dose prophylaxis and individualizing it to what is affordable for a given patient or nation, may bridge this gap created by global economic disparity. Greater efforts must be made in establishing comprehensive care and home care in developing nations in order for any type of prophylaxis to be successful. And finally maximizing the amount of time spent per week above target by using small but frequent doses of factor within one’s fiscal constraints may individualize prophylaxis treatment for greater returns in functional independence and quality of life.

## References

[1] Srivastava A, Brewer AK, Mauser-Bunschoten EP, Key NS, Kitchen S, Llinas A (2013). Guidelines for the management of hemophilia. Haemophilia.

[2] Nilsson IM, Berntorp E, Lofqvist T, Petterssont H (1992). Twenty-five years’ experience of prophylactic treatment in severe haemophilia A and B. J Intern Med.

[3] Gringeri A, Lundin B, Von Mackensen S, Mantovani L, Mannucci PM (2011). A randomized clinical trial of prophylaxis in children with hemophilia A ( the ESPRIT Study ). J Thromb Haemost.

[4] Manco-Johnson MJ, Abshire TC, Shapiro A, Riske B, Hacker MR, Kilcoyne R (2007). Prophylaxis versus episodic treatment to prevent joint disease in boys with severe hemophilia. N Engl J Med.

[5] Manco-Johnson MJ, Kempton CL, Reding MT, Lissitchkov T, Goranov S, Gercheva L (2013). Randomized, controlled, parallel-group trial of routine prophylaxis vs. on-demand treatment with sucrose-formulated recombinant factor VIII in adults with severe hemophilia A. J Thromb Haemost.

[6] Aledort LM, Haschmeyer RH, Pettersson H (1994). A longitudinal study of orthopaedic outcomes for severe fac tor-VIII-deficien t haemophiliacs. J Intern Med.

[7] Tagliaferri A, Feola G, Molinari AC, Santoro C, Rivolta GF, Cultrera DB (2015). Benefits of prophylaxis versus on-demand treatment in adolescents and adults with severe hemophilia A: the POTTER study. Thromb Haemost.

[8] Fischer K, Steen Carlsson K, Petrini P, Holmström M, Ljung R, van den Berg HM (2013). Intermediate-dose versus high-dose prophylaxis for severe hemophilia : comparing outcome and costs since the 1970s. Blood.

[9] Blanchette V, Israels S, Chan A, Rivard G, Cloutier S, Steele M (2014). Fifteen years of Canadian “tailored” prophylaxis: results from the Canadian Hemophilia Primary Prophylaxis Study (CHPS). Haemophilia.

[10] Molho P, Rolland N, Lebrun T, Dirat G, Courpied JP, Croughs T (2000). Epidemiological survey of the orthopaedic status of severe haemophilia A and B patients in France. Haemophilia.

[11] Ljung R, Aronis-Vournas S, Krunik-Auberger K, van den Berg M, Chambost H, Claeyssens S (2000). Treatment of children with haemophilia in Europe : a survey of 20 centres in 16 countries. Haemophilia.

[12] Blanchette VS, Mccready M, Achonu C, Abdolell M, Rivard G, Manco-Johnson MJ (2003). A survey of factor prophylaxis in boys with haemophilia followed in North American haemophilia treatment centres. Haemophilia.

[13] Risebrough N, Oh P, Blanchette V, Curtin J, Hitzler J, Feldman BM (2008). Cost-utility analysis of Canadian tailored prophylaxis, primary prophylaxis and on-demand therapy in young children with severe haemophilia A. Haemophilia.

[14] den Uijl IEM, Fischer K, van der Bom JG, Grobbee DE, Rosendaal FR, Plug I (2011). Analysis of low frequency bleeding data : the association of joint bleeds according to baseline FVIII activity levels. Haemophilia.

[15] Gringeri A, Ewenstein B, Reininger A (2014). The burden of bleeding in haemophilia : is one bleed too many ?. Haemophilia.

[16] Jansen NWD, Roosendaal G, Wenting MJG, Bijlsma JWJ, Theobald M, Hazewinkle HAW (2009). Very rapid clearance after a joint bleed in the canine knee cannot prevent adverse effects on cartilage and synovial tissue. Osteoarthr Cart.

[17] Oldenburg J (2015). Optimal treatment strategies for hemophilia : achievements and limitations of current prophylactic regimens. Blood.

[18] Chuansumrit A, Isarangkura P, Hathirat P (1995). Prophylactic treatment for hemophilia A patients: a pilot study. Southeast Asian J Trop Med Public Health.

[19] Wu R, Luke K-H, Poon M-C, Wu X, Zhang N, Zhao L (2011). Low dose secondary prophylaxis reduces joint bleeding in severe and moderate haemophilic children : a pilot study in China. Haemophilia.

[20] Tang L, Wu R, Sun J, Zhang X, Feng X, Zhang X (2013). Short-term low-dose secondary prophylaxis for severe / moderate haemophilia A children is beneficial to reduce bleed and improve daily activity, but there are obstacle in its execution : a multi-centre pilot study in China. Haemophilia.

[21] Verma SP, Dutta TK, Mahadevan S, Nalini P, Basu D, Biswal N (2016). A randomized study of very low-dose factor VIII prophylaxis in severe haemophilia – A success story from a resource limited country. Haemophilia.

[22] Hua B, Lian X, Li K, Lee A, Poon M-C, Zhao Y (2016). Low-dose tertiary prophylactic therapy reduces total number of bleeds and improves the ability to perform activities of daily living in adults with severe haemophilia A : a single- centre experience from Beijing. Blood Coagul Fibrinolysis.

